# The association between cesarean birth and breastfeeding initiation in Odisha, India: A mother fixed effects analysis

**DOI:** 10.1371/journal.pone.0287796

**Published:** 2024-02-12

**Authors:** Smita, Nathan Franz, Diane Coffey

**Affiliations:** 1 Amity Institute of Social Sciences, Amity University, Noida, Uttar Pradesh, India; 2 Department of Economics, University of Texas at Austin, Austin, Texas, United States of America; 3 Population Research Center, University of Texas at Austin, Austin, Texas, United States of America; 4 r.i.c.e., a Research Institute for Compassionate Economics, Amston, Connecticut, United States of America; University of Botswana, BOTSWANA

## Abstract

Cesarean births are becoming more common in India, with health implications for both mothers and infants. Between 2005 and 2015, the proportion of cesarean births to total births in India roughly doubled, from 9% to 17%. We analyze Annual Health Survey data from the state of Odisha in eastern India. These population-level, longitudinal data on births between 2007 and 2011 allows us to estimate the association between cesarean birth and breastfeeding outcomes using mother fixed effects. Mother fixed effects allow comparisons of siblings born to the same mother who experienced different types of births (vaginal and cesarean). This empirical strategy controls for many potential observable and unobservable confounders in the relationship. Ordinary Least Squares linear probability models without mother fixed effects find that babies born by cesarean in Odisha are about 14 percentage points (*p*<0.001) more likely to experience delayed initiation of breastfeeding (that is, not being breastfed in the first 24 hours) compared with babies born vaginally. After introducing mother fixed effects, we find that babies born by cesarean are 11 percentage points more likely to (*p*<0.001) experience delayed initiation of breastfeeding. Because breastfeeding success is important for protecting against infectious disease in this context, future research should investigate whether cesarean birth impacts other aspects of breastfeeding as well.

## 1. Introduction

Globally, the proportion of cesarean births to total births has increased from about 7% in 1990 to about 19% in 2014 [1]. National Family Health Survey (NFHS) data show that India’s cesarean rate increased from about 3% of births in 1992–3 to about 17% in 2015 [2,3]. The average rate of cesarean births in 2015 masks large variation across states: Nagaland and Bihar have the lowest cesarean rates (about 6%), while Andhra Pradesh and Telangana have the highest rates (40% and 58% respectively). The cesarean rate in the state of Odisha, which this paper studies, is 14% [3].

The consequences of India’s recent increase in cesarean birth for maternal and child health and nutrition merit careful investigation. Expanding access to cesarean birth may save lives in situations of fetal distress, eclampsia, placenta previa, breech or transverse presentation, and other medical indications. Indeed, the WHO estimates that, at the population level, cesarean birth may be medically justified for between 10 and 15 percent of births [4]. However, unnecessary cesareans pose risks to both mothers and infants, including longer recovery times and increased risk of infection for mothers and respiratory complications for newborns [5,6].

Prior studies from both high- and low-resource settings, including Mexico, Puerto Rico, Australia, Taiwan, Italy, Ethiopia, and India, have found negative associations between cesarean birth and breastfeeding outcomes [7–15]. Among the reasons researchers believe these associations exist are that the anesthesia used for cesarean births may make the baby less alert, that the infant’s respiratory function may be impacted by the mode of birth, that postoperative care might reduce mother-infant interaction, including skin-to-skin contact, and that mothers recovering from cesarean births may find it less comfortable to breastfeed [10,13,16].

This study investigates the consequences of cesarean birth on breastfeeding initiation in Odisha, a low-income state in India. Specifically, we model whether breastfeeding was started in the first 24 hours as a function of type of birth. Initiation of breastfeeding in the first 24 hours is a widely recognized to help establish a mother’s milk production, which is critical to the success of the breastfeeding relationship. In India, 55% of babies are exclusively breastfed for the first six months; in Odisha, this figure is 65% [3].

Successful breastfeeding is linked to the development of a child’s immunity and to her nutrition [17]. It is especially important in the Indian context because of India’s high infectious disease burden [18,19]. Despite strong cultural support for breastfeeding in India and advocacy efforts from organizations such as the National Breastfeeding Committee, the Public Health Resource Network, and the Breastfeeding Network Promotion of India, there is still substantial opportunity to improve breastfeeding practices, and especially to increase the proportion of newborns who are fed colostrum.

Our study uses data from the state of Odisha, a relatively poor state in eastern India where the government has recently made several policy changes meant to improve child health and nutrition [20]. The NFHS-2015 found that the fraction of children breastfed in the first hour in Odisha was about 70%, which is higher than for India as a whole. However, Odisha’s burdens of poor child health are similar to India’s: the NFHS-2015 found about 34% of children under five are *stunted* and 20% are *wasted*, compared to 38% and 21% respectively for India as a whole [3]. Stunting is height-for-age that is more than 2 SD below the WHO’s reference norms for healthy children; wasting is weight-for-height that is more than 2 SD below the norms. The infant mortality rate for both Odisha and India were about 40 per 1000 live births [3]. Because sanitation is worse in Odisha (65% of households defecate in the open without a toilet or latrine) than in India as a whole (40% of households defecate in the open), breastfeeding may be even more important to child health in Odisha than in places with less open defecation [3].

Although these are strong reasons to believe that cesarean birth may have a causal impact on some breastfeeding outcomes, a limitation of the existing literature is that it does not allow researchers to control for unobserved mother-level characteristics that may confound the relationship between type of birth and breastfeeding outcomes. Unobserved mother-level characteristics may be especially important in the Indian context, where mothers with higher socioeconomic status (SES) may be more likely to deliver by cesarean and to delay initiation of breastfeeding.

Indeed, S1 Fig in the Supporting Information shows that in the state of Odisha more educated mothers are both more likely to have cesarean births and to delay breastfeeding. This may be surprising because education usually promotes breastfeeding practices. However, in Odisha, as in much of India, there is a long tradition of discarding colostrum. Indeed, Devi & Behera’s study [21] of breastfeeding practices in southern Odisha found that over 80% of mothers discarded colostrum. The remaining mothers did not discard it only because they “had no clear views of it” (p. 754), not because they knew it to be good for their babies. Although there is little Odisha-specific research on breastfeeding initiation, studies from other parts of India support the idea that late initiation is often considered desirable [22,23] and that the highest SES women are the most likely to be expected to delay breastfeeding until ceremonies involving washing the breasts or gift exchanges can occur [24]. Given that maternal SES is correlated with both cesarean birth and delayed initiation of breastfeeding, estimates of the effect of cesarean birth that do not control for unobserved characteristics of the mother may be upwardly biased.

Our study adds to the existing literature by using longitudinal data from India’s Annual Health Survey (AHS) that permit a mother fixed effects analysis that controls for any time-invariant characteristics that are shared across siblings. Specifically, the AHS data allow us to ask: Are children who were delivered by cesarean less likely to be breastfed in the first 24 hours than siblings born vaginally?

This study finds further evidence of the association between cesarean birth and breastfeeding outcomes in a new context, and it makes the new contribution of controlling for both observed and unobserved characteristics of the child’s mother and household. We note, however, that a limitation of the study is that it cannot account for differences in the infant’s ability to breastfeed, which may be impacted by the mode of birth [16]. Therefore, we are unable to definitively pinpoint the mechanisms through which the relationship between mode of birth and delayed initiation of breastfeeding operate in this context.

In this article, we use National Family Health Survey (NFHS) 2015 data for the purpose of describing the setting and we use the AHS data for our analyses. The NFHS is India’s Demographic and Health Survey; its data are more recent but it does not allow mother fixed effects analysis.

## 2. Methods

### 2.1 Data

Our analyses use data from the Annual Health Survey (AHS), which was conducted by the Office of the Registrar General & Census Commissioner of India in three rounds between 2010 and 2012. It was commissioned by the Ministry of Health and Family Welfare. The 2010 round collected data on births between 2007 and 2009; the 2011 round collected data on births in 2010; and the 2012 round collected data on births in 2011. We use all three rounds of the Woman Pregnancy Schedule (WPS) for the state of Odisha, which are publicly available online [25]. Respondents included all ever-married women who reported pregnancy in the reference period.

The AHS is a sample survey that was designed to provide representative estimates of infant mortality at the district level. The Office of the Registrar General & Census Commissioner of India’s Annual Health Survey Report (2015) [26] describes the AHS sample design as a “uni-stage stratified simple random sample without replacement” (p. 6). The three strata within each district are urban areas, small villages (less than 2000 people as per the 2001 Census), and large villages (2000 or more people as per the 2001 Census). Our analyses use sample weights to account for the stratified design.

To select respondents within each district, the AHS first selected primary sampling units (PSUs), which were Census Enumeration Blocks in urban areas and villages in rural areas. Villages with populations of 2000 or more (as per the 2001 Census) were divided into geographically contiguous segments that did not exceed 2,000 population. Then one of the segments was randomly selected for inclusion in the survey. Enumerators performed a household listing and randomly selected households to be interviewed. In the second and third rounds of the survey, households from the first round were revisited. New households were added in the second and third rounds to maintain a similar sample size across rounds.

S1 Table in the Supporting Information summarizes the number of observations in each round of the AHS and the number that are matched to siblings in other rounds (and are therefore used to identify effects of cesarean birth in mother fixed effects regressions). In the WPS data, observations can be matched across rounds when the child’s mother was both re-interviewed by the AHS and had a subsequent birth. Unfortunately, the data do not distinguish between mothers who are not included in subsequent AHS rounds because they did not have another child and those who were lost to follow up.

### 2.2 Measurement of the dependent and independent variables

#### 2.2.1 Independent variable

The independent variable of interest in this study is type of birth. The AHS provides information on whether the birth is vaginal, assisted (with vacuum or forceps) vaginal, or cesarean. We combine vaginal and assisted vaginal deliveries into a single category and regress breastfeeding outcomes on a binary indicator for whether the birth was cesarean.

#### 2.2.2 Dependent variable

Our dependent variable of interest is an indicator variable for whether breastfeeding was not initiated in the first 24 hours, as reported by the mother.

#### 2.2.3 Control variables

In the controlled Ordinary Least Squares (OLS) regression (specifications without mother fixed effects), we include indicators for the child’s mother’s education (9 categories) and for her household’s socioeconomic status. In particular, we control for indicators for the household’s lighting source (electricity (reference category), kerosene, and other), type of cooking fuel (firewood (reference category), crop residue, cow dung, coal, liquid petroleum gas (LPG), other), and ownership of each of the following assets: toilet/latrine, radio, television, computer, washing machine, refrigerator, sewing machine, bicycle, scooter, car, tractor, and water pump. These control variables are dropped from mother fixed effects analyses because they were collected only the first time the household was interviewed and are therefore the same across siblings, which means that they are absorbed in the mother fixed effect. We further include control variables for the child’s birth order because some child health outcomes are correlated with higher birth order among siblings in India [27]. In order to control for possible time trends in reporting of initiation of breastfeeding (which might be present if the Government of Odisha were promoting breast-feeding more or less at different times) we control for survey round.

### 2.3 Statistical analysis

Section 3.2 presents OLS linear probability models (uncontrolled and controlled) showing how cesarean births predict initiation of breastfeeding (N = 123,823). An equation for the controlled model is given in the Supporting Information. Section 3.2 also presents the results of a mother fixed effects model that is estimated using the STATA command *reghdfe* (N = 47,814) [28]. The sample size for these results is smaller than for the controlled model because the fixed effects models only include those children who have a sibling who was also included in the survey. The coefficient on birth type in this model is estimated off of the 4,379 children who had a different type of birth than they did. We note that vaginal birth after cesarean is not very common in Odisha; only 11% of births after cesarean are vaginal. Most of the sibling pairs from whom the fixed effects estimates are made are births for which a lower birth order child had a vaginal birth, and a higher birth order child has a cesarean birth. Information on the other 43,433 children is used to estimate coefficients on control variables. Clustered standard errors for coefficients in the mother fixed effects models are estimated; the child’s PSU is the cluster variable.

To demonstrate the robustness of the results to different modeling choices, we present results of a fixed effects logit model in the Supporting Information. In this model, only those children who are matched to a sibling and for whom the siblings’ types of birth differ are included in the regression [29]. Controls that do not vary across siblings are omitted.

## 3. Results

### 3.1 Descriptive statistics

Table 1 presents summary statistics, by survey round, of the independent and dependent variables of interest. It shows a statistically significant increase in cesarean birth across the three rounds of the survey, from 8.8% of children born between 2007 and 2009 to 11.2% of children born in 2011. This is an increase of more than 25% in only a few years. There was also a small decline in the fraction of births for which breastfeeding was not initiated in the first 24 hours, from 6.2% of births between 2007 and 2009, to 4.3% of births in 2011. These differences may reflect real or reporting differences induced by the Odisha government’s attempts to promote breastfeeding. They may also reflect differences across rounds in how interviewers were trained to ask this question. The controls for survey round in our regression models mitigate concerns about reporting breastfeeding outcomes.

**Table 1:** Summary statistics for variables of interest, by survey round, N = 132,816.

	2007–09		2010		2011	
	weighted %	n	weighted %	n	weighted %	n
**independent variable**						
cesarean birth	8.8	7051	10.1	2546	11.2	4526
**dependent variable**						
delayed initiation of breastfeeding	6.2	5050	5.0	1252	4.3	1636
**N**	75,047		20,007		28,769	

Note: The statistics in the table summarize data from each of the three rounds of the Annual Health Survey conducted in Odisha between 2010 and 2012. Survey weights are used for the percentages in each column.

Table 2 presents summary statistics for the control variables used in our regression models. It pools data from across rounds to summarize the distribution of birth order and mother’s education and to show the fraction of households that own various assets and that use different types of cooking fuel and lighting. The modal child is the mother’s first live birth, and each higher birth order is less common than the preceding one. The modal level of education among mothers is middle school; most households do not have LPG for cooking but do have electricity for lighting.

**Table 2:** Summary statistics for time-invariant control variables and birth order, N = 123,825.

	full sample (2007–11)	
	weighted %	n
**mother’s education**		
illiterate	26	27493
literate without formal education	7	8783
below primary	11	13698
primary	14	18537
middle	22	28098
secondary/matric (class 10)	11	14082
higher secondary (class 12)	5	6400
graduate	4	5880
post-graduate	1	857
**household asset ownership**		
toilet	23	29535
radio	11	13643
television	31	41897
computer	3	4287
washing machine	2	3429
refrigerator	8	10808
sewing machine	5	6894
bicycle	66	82970
scooter	15	20173
car	2	2422
tractor	1	1161
water pump	6	7363
**cooking fuel**		
firewood	68	82251
crop residue	9	10767
cow dung	6	7652
coal/lignite/charcoal	2	4882
LPG/PNG	8	7983
other	8	10293
**lighting source**		
electricity	49	68283
kerosene	50	54651
other	1	894
**birth order**		
1	43	53790
2	31	38342
3	15	17655
4	7	7792
5 or higher	5	6246

Note: The statistics in the table summarize data from all three rounds of the Annual Health Survey conducted in Odisha between 2010 and 2012. Survey weights are used for the percentages in each column.

S2 Table in the Appendix compares mothers who had more than one birth recorded by the AHS to mothers in the three AHS survey rounds who only one birth recorded in the AHS. This is relevant because only mothers who had more than one birth are eligible for the fixed effects sample. Mothers who had more than one birth are similar in age, but less educated and more likely to be from a disadvantaged social group.

### 3.2 Results from uncontrolled, controlled, and mother fixed effects regression models

Table 3 presents our main results on initiation of breastfeeding. Coefficients from OLS linear probability model regressions are presented; clustered standard errors are shown in parentheses. The results in Column (1) show the uncontrolled association between cesarean birth and delayed initiation of breastfeeding. Children born by cesarean were 13.8 percentage points more likely to have delayed initiation of breastfeeding compared to children born vaginally. Column (2) adds controls for survey rounds, maternal education, and household economic status. In the fully controlled model (without mother fixed effects) shown in Column (2), babies born by cesarean are 14.1 percentage points more likely to experience delayed initiation of breastfeeding. Finally, Column (3) shows results using mother fixed effects. Here, the coefficient on cesarean section is smaller in magnitude than for the other models but is nevertheless statistically significantly different from zero. Compared to siblings who were born vaginally, children born by cesarean are 10.9 percentage points more likely to experience delayed initiation of breastfeeding.

**Table 3:** OLS linear probability models of delayed initiation of breastfeeding on cesarean birth, weighted.

	(1)				(2)^a^				(3)^b^		
**dependent variable:**	delayed initiation of breastfeeding										
**model type:**	OLS LPM								fixed effects OLS LPM		
**n**	132,821				123,823				47,814		
	β	*P*-val	95% CI		β	*P*-val	95% CI		β	*P*-val	95% CI
cesarean birth	0.138	0.000	[0.128, 0.147]		0.141	0.000	[0.131, 0.152]		0.109	0.000	[0.092, 0.126]
**survey round**											
born 2007–09					.	.	.		.	.	.
born 2010					-0.014	0.000	[-0.020, -0.009]		-0.015	0.002	[-0.025, -0.006]
born 2011					-0.023	0.000	[-0.030, -0.016]		-0.019	0.000	[-0.029, -0.009]
**mother FE**											

Note: *P*-values are shown in parentheses and 95% confidence intervals are shown in brackets with standard errors clustered at the PSU level. Weights are used in each regression.

^a^ Controlling for survey round, birth order, mother’s education level, cooking fuel used, lighting used, and ownership of toilet, radio, TV, computer, washing machine, refrigerator, sewing machine, bicycle, scooter, care, and water pump.

^b^ Controlling for survey round, birth order, and mother fixed effects.

These results use data on all births which survived to the time of the survey. Initiation of breastfeeding was only collected for about half of the 4% of live births who subsequently died. S3 Table in the Supporting Information repeats the OLS linear probability models with all the available data (including children who died). Including these additional observations does not change our results. S4 Table in the Supporting Information presents a robustness check of the results using logistic regression models. (These models do not use weights because fixed effects logit does not permit the use of weights.) These models find similar results to the weighted results presented here.

## 4. Discussion

### 4.1 Implications of the results

This paper joins a robust prior literature that finds impacts of cesarean birth on breastfeeding outcomes [7,9–12,15]. These papers use cross-sectional data to document negative associations between cesarean birth and breastfeeding. In addition to finding similar results to these papers in the cross-sectional analysis, this paper also uses a mother fixed effects strategy to estimate the association between cesarean birth and initiation of breastfeeding within sibling pairs. This empirical strategy is useful in the context of Odisha, where higher SES mothers are both more likely to give birth by cesarean and to delay initiation of breastfeeding.

The fact that babies in Odisha who are born by cesarean are between 10 and 15 percentage points less likely to be breastfed in the first 24 hours of life relative to babies born vaginally may have important implications for their health. The WHO recommends initiation of breastfeeding as early as the first hour in order to promote a mother’s milk supply and to reduce the use of supplemental milks later in infancy [17]. In a context such as Odisha where supplementing babies’ diets with non-human milk can lead to intestinal disease, delaying initiation of breastfeeding may have wide-reaching impacts. Health care providers should be aware that mothers may need additional encouragement to initiate breastfeeding in contexts where discarding colostrum is common and that mothers who have had cesarean births may need additional support for initiation of breastfeeding.

### 4.2 Limitations of the study

One important limitation of this study is that the most recent births are from 2011. The government of Odisha has invested in early life health in ways that may have changed the relationship between cesarean birth and delayed initiation of breastfeeding since then. It is unfortunately not possible to repeat our analyses with more recent data because more recent data sources such as the NFHS do not collect information on initiation of breastfeeding for multiple children born to the same mother.

The fixed effects analyses are necessarily limited by the fact that only some of the mothers have more than one birth that is observed during the period of the AHS. The fact that the fixed effects sample, which over-represents less advantaged mothers relative the full sample, finds a negative association between initiation of breastfeeding and cesarean birth, suggests that some of the physical aspects of cesarean birth, which affect both the mother and the baby, may be important mechanisms for breastfeeding outcomes.

The study is also limited in its ability to analyze breastfeeding outcomes other than initiation of breastfeeding using a mother fixed effects strategy. Although the AHS collects data on the timing of introduction of animal milk or formula milk, for example, these outcomes do not lend themselves to mother fixed analysis in the same way that initiation of breastfeeding does. A concern that has emerged in the recent methodological literature is that estimation using high-dimensional fixed effects—such as mother fixed effects—can suffer from a Selection into Identification challenge [27,30]. In particular, as mentioned above, effects in mother fixed effects models are identified from siblings who have different types of births. These may be a select group. Moreover, if we were studying a dependent variable that applied to different ages for different observations, then controlling for the age of the child at the time of the reference period would be important but difficult to combine with mother fixed effects without threatening causal identification.

Although Selection into Identification would generate important concerns for some measures of breastfeeding quality, they are mitigated for the *delayed initiation* of breastfeeding, which we study in this paper, for two reasons. First, our dependent variable is age-specific: we study initiation of breastfeeding at the uniform age of the first day of life. Second, our identification is unlikely to be threatened by endogenous duration of lactational amenorrhea because we study delayed initiation of breastfeeding, not breastfeeding behaviors that are known to predict parity progression. However, future research might usefully investigate whether initiation of breastfeeding predicts birth spacing in this context.

## 5. Conclusion

Our study finds that babies born by cesarean in the Indian state of Odisha are significantly more likely to experience delayed (>24 hours) initiation of breastfeeding. We arrive at this conclusion using a mother fixed effects study design that addresses concerns about endogenous selection into type of birth. In short, we draw similar conclusions whether we make across-family or within-family comparisons of babies born by cesarean to those who were born vaginally. These findings are important because timing of initiation of breastfeeding may impact other breastfeeding behaviors and child health. Future research could usefully study relationships between cesarean birth and other breastfeeding behaviors in Odisha and in the rest of India.

## Supporting information

S1 Fig Motivation for mother fixed effects models: Better educated mothers in Odisha are both more likely to deliver by cesarean and to delay initiation of breastfeeding. (DOCX)

S1 Table Observations in all three rounds of the Annual Health Survey in Odisha. (DOCX)

S2 Table Comparison of mothers who had one birth to those who are included in the fixed effects sample. (DOCX)

S3 Table Replication of Table 3 (OLS linear probability models of delayed initiation of breastfeeding on cesarean birth), including babies who died. (DOCX)

S4 Table Logit models of delayed initiation of breastfeeding on cesarean birth. (DOCX)

S1 File Equation for OLS Linear Probability Model in Column 2 of Table 3. (DOCX)
