# Supplementary material for: The association between cesarean birth and breastfeeding initiation in Odisha, India: A mother fixed effects analysis
Source: PLoS One. 2024 Feb 12;19(2):e0287796. doi: 10.1371/journal.pone.0287796 (PMC10861043; doi:10.1371/journal.pone.0287796)
Supplement: S1 Fig — (DOCX) [file pone.0287796.s001.docx]

*Figure S1. Motivation for mother fixed effects models: Better educated mothers in Odisha are both more likely to deliver by cesarean and to delay initiation of breastfeeding*

*Panel A. Proportion of births by cesarean*

*Panel B. Proportion of births for which initiation of breastfeeding was delayed (>24 hours)*

Note: Graphs show weighted proportions and 95% CIs for each education subgroup.
