# Supplementary material for: The association between cesarean birth and breastfeeding initiation in Odisha, India: A mother fixed effects analysis
Source: PLoS One. 2024 Feb 12;19(2):e0287796. doi: 10.1371/journal.pone.0287796 (PMC10861043; doi:10.1371/journal.pone.0287796)
Supplement: S1 Table — (DOCX) [file pone.0287796.s002.docx]

*Table S1. Observations in all three rounds of the Annual Health Survey in Odisha*

|  | **number** |
| --- | --- |
| **round 1 (live births & still births between 2007 and 2009)** |  |
| total observations | 83,490 |
| observations which are surviving births | 75,199 |
| observations matched to a sibling in round 2, but not 3 | 4,320 |
| observations matched to a sibling in round 3, but not 2 | 6,774 |
| observations matched to siblings in both rounds 2 and 3 | 396 |
|  |  |
| **round 2 (live births & still births in 2010)** |  |
| total observations | 25,664 |
| observations which are surviving births | 22,543 |
| observations matched to a sibling in round 1, but not 3 | 4,072 |
| observations matched to a sibling in round 3, but not 1 | 1,172 |
| observations matched to siblings in both rounds 1 and 3 | 361 |
|  |  |
| **round 3 (live births & stillbirths in 2011)** |  |
| total observations | 39,333 |
| observations which are surviving births | 35,135 |
| observations matched to a sibling in round 1, but not 2 | 6,258 |
| observations matched to a sibling in round 2, but not 1 | 1,167 |
| observations matched to siblings in both rounds 1 and 3 | 373 |

*Note: Observations can be matched to siblings in subsequent rounds when the child’s mother was both re-interviewed by the AHS and had a subsequent birth.*
