# Supplementary material for: The association between cesarean birth and breastfeeding initiation in Odisha, India: A mother fixed effects analysis
Source: PLoS One. 2024 Feb 12;19(2):e0287796. doi: 10.1371/journal.pone.0287796 (PMC10861043; doi:10.1371/journal.pone.0287796)
Supplement: S2 Table — (DOCX) [file pone.0287796.s003.docx]

*Table S2. Comparison of mothers who had one birth to those who are included in the fixed effects sample*

|  | **mothers with 1 birth** | | **mothers with >1 birth** | |
| --- | --- | --- | --- | --- |
|  | weighted mean | n | weighted mean | n |
| **number of children** | **1** | 85,006 | 2.5 | 47,812 |
| **mother’s age (years)** | **26.7** | 85,006 | 26.6 | 47,812 |
| **mother's education** |  |  |  |  |
| illiterate (%) | 21 | 15,232 | 34 | 13,790 |
| literate without formal education (%) | **7** | 5,446 | 8 | 3,807 |
| below primary (%) | 10 | 8,924 | 11 | 5,618 |
| primary (%) | 15 | 12,754 | 14 | 7,237 |
| middle (%) | 24 | 20,570 | 19 | 9,636 |
| secondary/matric (class 10) (%) | 12 | 10,668 | 8 | 4,410 |
| higher secondary (class 12) (%) | 6 | 5,219 | 3 | 1,697 |
| graduate (%) | 5 | 5,074 | 2 | 1,287 |
| post-graduate (%) | 1 | 775 | 0 | 181 |
| **caste** |  |  |  |  |
| Scheduled Caste (%) | 19 | 16,358 | 22 | 10,886 |
| Scheduled Tribe (%) | 22 | 16,914 | 32 | 13,451 |
| other group (%) | 59 | 51,501 | 47 | 23,346 |
| **Hindu (%)** | 95 | 80,705 | 95 | 45,101 |

*Note: The “mothers with 1 birth” column include observations that observe whether breastfeeding was initiated late, whether the birth was cesarean, and the order of birth, but which are not included in the fixed effects regression sample. The “mothers with >1 birth” column include observation that are in the fixed effects regression sample. Survey weights are used for the means in each column.*
