# Supplementary material for: The association between cesarean birth and breastfeeding initiation in Odisha, India: A mother fixed effects analysis
Source: PLoS One. 2024 Feb 12;19(2):e0287796. doi: 10.1371/journal.pone.0287796 (PMC10861043; doi:10.1371/journal.pone.0287796)
Supplement: S3 Table — (DOCX) [file pone.0287796.s004.docx]

*Table S3. Replication of Table 3 (OLS linear probability models of delayed initiation of breastfeeding on cesarean birth), including babies who died*

|  | (1) | | |  | (2)^a^ | | |  | (3)^b^ | | |
| --- | --- | --- | --- | --- | --- | --- | --- | --- | --- | --- | --- |
| **dependent variable:** | delayed initiation of breastfeeding | | | | | | | | | | |
| **model type:** | OLS LPM | | | | | | |  | fixed effects OLS LPM | | |
| **n** | 135,066 | | |  | 125,676 | | |  | 49,556 | | |
|  | β | *P*-val | 95% CI |  | β | *P*-val | 95% CI |  | β | *P*-val | 95% CI |
| cesarean | 0.138 | 0.000 | [0.128, 0.147] |  | 0.142 | 0.000 | [0.131, 0.152] |  | 0.110 | 0.000 | [0.093, 0.128] |
| **survey round** |  |  |  |  |  |  |  |  |  |  |  |
| born 2007-09 |  |  |  |  | . | . | . |  | . | . | . |
| born 2010 |  |  |  |  | -0.005 | 0.029 | [-0.010, -0.001] |  | -0.004 | 0.382 | [-0.013, 0.005] |
| born 2011 |  |  |  |  | -0.014 | 0.000 | [-0.020, -0.008] |  | -0.014 | 0.004 | [-0.024, -0.005] |
| **mother FE** |  |  |  |  |  |  |  |  | ✓ | | |

Note: P-values are shown in parentheses and 95% confidence intervals are shown in brackets with standard errors clustered at the PSU level. Weights are used in each regression.

^a^ Controlling for survey round, birth order, mother’s education level, cooking fuel used, lighting used, and ownership of toilet, radio, TV, computer, washing machine, refrigerator, sewing machine, bicycle, scooter, care, and water pump.

^b^ Controlling for survey round, birth order, and mother fixed effects.
