# Supplementary material for: The association between cesarean birth and breastfeeding initiation in Odisha, India: A mother fixed effects analysis
Source: PLoS One. 2024 Feb 12;19(2):e0287796. doi: 10.1371/journal.pone.0287796 (PMC10861043; doi:10.1371/journal.pone.0287796)
Supplement: S4 Table — (DOCX) [file pone.0287796.s005.docx]

*Table S4. Logit models of delayed initiation of breastfeeding on cesarean birth*

|  | (1) | | |  | (2)^a^ | | |  | (3)^b^ | | |
| --- | --- | --- | --- | --- | --- | --- | --- | --- | --- | --- | --- |
| **dependent variable:** | delayed initiation of breastfeeding | | | | | | | | | | |
| **model type:** | logit OR | | | | | | |  | fixed effects logit OR | | |
| **n** | 132,821 | | |  | 123,820 | | |  | 4,379 | | |
|  | β | *P*-val | 95% CI |  | β | *P*-val | 95% CI |  | β | *P*-val | 95% CI |
| cesarean | 4.967 | 0.000 | [4.665, 5.288] |  | 5.458 | 0.000 | [5.087, 5.855] |  | 4.456 | 0.000 | [3.164, 6.275] |
| **survey round** |  |  |  |  |  |  |  |  |  |  |  |
| born 2007-09 |  |  |  |  | . | . | . |  | . | . | . |
| born 2010 |  |  |  |  | 0.749 | 0.000 | [0.686, 0.817] |  | 0.671 | 0.001 | [0.532, 0.845] |
| born 2011 |  |  |  |  | 0.595 | 0.000 | [0.537, 0.659] |  | 0.543 | 0.000 | [0.429, 0.686] |
| **mother FE** |  |  |  |  |  |  |  |  | ✓ | | |

Note: P-values are shown in parentheses and 95% confidence intervals are shown in brackets with standard errors clustered at the PSU level. In column 3, the fixed effects logit model, standard errors are computed using a cluster bootstrap, where the PSU is the cluster. Weights are not used in these regressions.

^a^ Controlling for survey round, birth order, mother’s education level, cooking fuel used, lighting used, and ownership of toilet, radio, TV, computer, washing machine, refrigerator, sewing machine, bicycle, scooter, care, and water pump.

^b^ Controlling for survey round, birth order, and mother fixed effects.
