# Supplementary material for: The association between cesarean birth and breastfeeding initiation in Odisha, India: A mother fixed effects analysis
Source: PLoS One. 2024 Feb 12;19(2):e0287796. doi: 10.1371/journal.pone.0287796 (PMC10861043; doi:10.1371/journal.pone.0287796)
Supplement: S1 File — (DOCX) [file pone.0287796.s006.docx]

*Equation S1. Equation for OLS Linear Probability Model in Column 2 of Table 3.*

The following equation corresponds to the fully controlled model without mother fixed effects in Column 2 of Table 3 of the main text (N=123,823):

$$\begin{aligned} {delayed initation}_{i}=\beta_{0}+{\beta_{1}cesarean section_{i}+ \alpha}_{i}+R_{i}\Phi+ E_{i}\Theta+ X_{i}\Gamma+\epsilon_{i}. \#\left( 1 \right) \end{aligned}$$

${delayed initiation}_{i}$ is an indicator that breastfeeding was initiated late (>24 hours) or not initiated at all. $cesarean section_{i}$ is an indicator for whether child *i* was born by cesarean. $\alpha_{i}$ is a survey round fixed effect which indicates whether the child was born between 2007 and 2009, in 2010, or in 2011. $R_{i}$ is a vector of indicators for the child’s birth order, censored at 5+. $E_{i}$ is a vector of indicators for the mother’s level of education. $X_{i}$ is a vector of indicators for household assets ownership, cooking fuel type, and lighting type.
